# Adenoviral Vectors as Vaccines for Emerging Avian Influenza Viruses

**DOI:** 10.3389/fimmu.2020.607333

**Published:** 2021-01-29

**Authors:** Lucas J. Kerstetter, Stephen Buckley, Carly M. Bliss, Lynda Coughlan

**Affiliations:** ^1^ Department of Microbiology and Immunology, University of Maryland School of Medicine, Baltimore, MD, United States; ^2^ Division of Cancer & Genetics, Division of Infection & Immunity, School of Medicine, Cardiff University, Wales, United Kingdom; ^3^ Center for Vaccine Development and Global Health, University of Maryland School of Medicine, Baltimore, MD, United States

**Keywords:** adenovirus, adenoviral vector, vaccine, immunogenicity, influenza, avian influenza, highly pathogenic, highly pathogenic avian influenza

## Abstract

It is evident that the emergence of infectious diseases, which have the potential for spillover from animal reservoirs, pose an ongoing threat to global health. Zoonotic transmission events have increased in frequency in recent decades due to changes in human behavior, including increased international travel, the wildlife trade, deforestation, and the intensification of farming practices to meet demand for meat consumption. Influenza A viruses (IAV) possess a number of features which make them a pandemic threat and a major concern for human health. Their segmented genome and error-prone process of replication can lead to the emergence of novel reassortant viruses, for which the human population are immunologically naïve. In addition, the ability for IAVs to infect aquatic birds and domestic animals, as well as humans, increases the likelihood for reassortment and the subsequent emergence of novel viruses. Sporadic spillover events in the past few decades have resulted in human infections with highly pathogenic avian influenza (HPAI) viruses, with high mortality. The application of conventional vaccine platforms used for the prevention of seasonal influenza viruses, such as inactivated influenza vaccines (IIVs) or live-attenuated influenza vaccines (LAIVs), in the development of vaccines for HPAI viruses is fraught with challenges. These issues are associated with manufacturing under enhanced biosafety containment, and difficulties in propagating HPAI viruses in embryonated eggs, due to their propensity for lethality in eggs. Overcoming manufacturing hurdles through the use of safer backbones, such as low pathogenicity avian influenza viruses (LPAI), can also be a challenge if incompatible with master strain viruses. Non-replicating adenoviral (Ad) vectors offer a number of advantages for the development of vaccines against HPAI viruses. Their genome is stable and permits the insertion of HPAI virus antigens (Ag), which are expressed *in vivo* following vaccination. Therefore, their manufacture does not require enhanced biosafety facilities or procedures and is egg-independent. Importantly, Ad vaccines have an exemplary safety and immunogenicity profile in numerous human clinical trials, and can be thermostabilized for stockpiling and pandemic preparedness. This review will discuss the status of Ad-based vaccines designed to protect against avian influenza viruses with pandemic potential.

## Introduction

**Figure 1:**
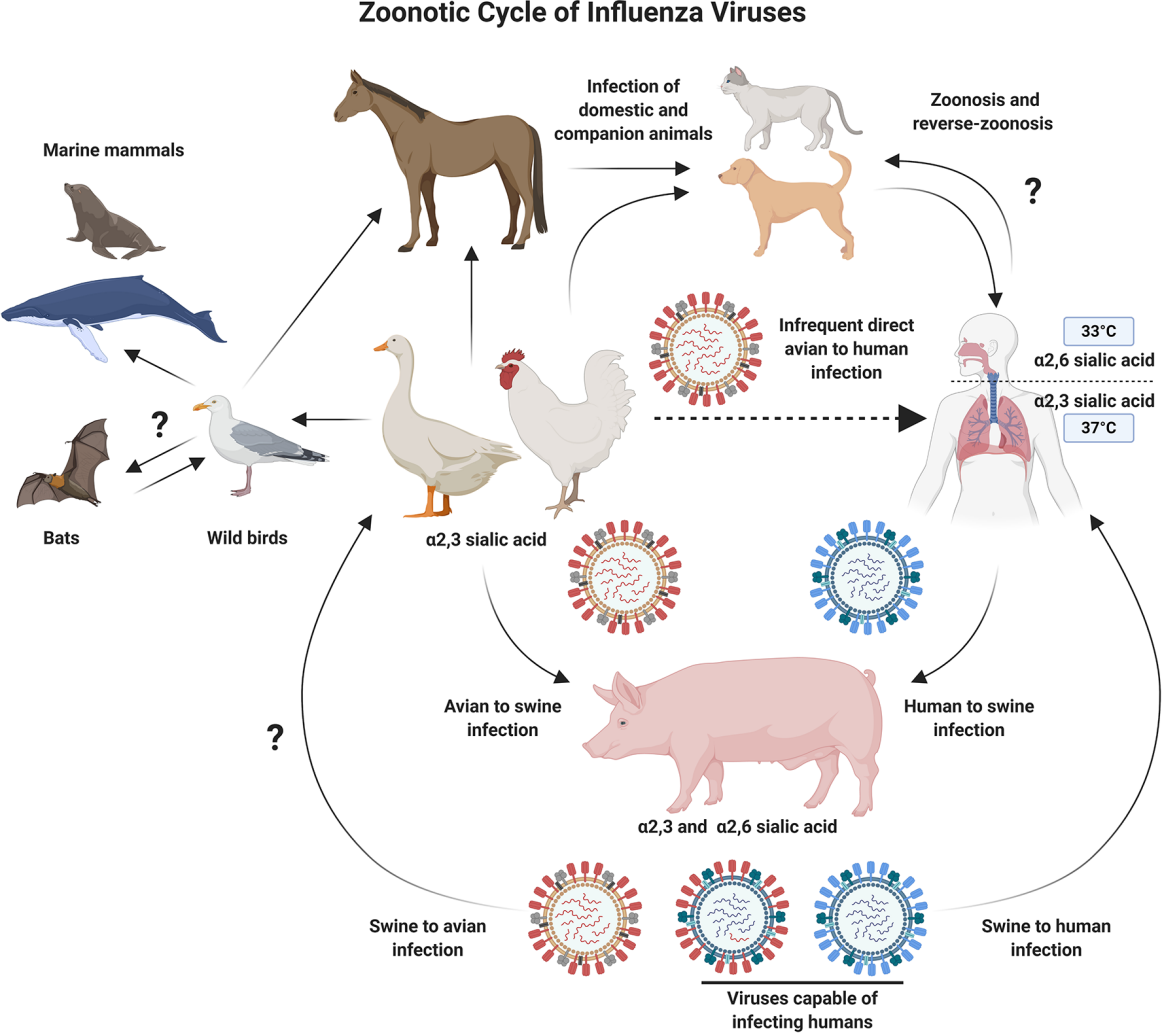
Schematic Diagram Showing Zoonotic Cycle of Influenza Viruses. Influenza A viruses can infect multiple animal species, which increases the probability of cross-species transmission events. Migratory and aquatic birds represent natural reservoirs for avian influenza viruses, and pigs act as a mixing vessel, allowing the reassortment of diverse influenza viruses. The process of reassortment could lead to the emergence of novel influenza subtypes which are better adapted for infection and transmission in humans. Several barriers to this process also exist, including, but not limited to receptor usage preferences. Direct infection of humans with avian influenza viruses is an infrequent event. However, the potential for adaptation while maintaining high pathogenicity is a major concern and drives efforts to develop improved vaccines against emerging avian influenza viruses. Figure created with ^©^BioRender - Biorender.com.

Influenza viruses belong to the family *Orthomyxoviridae* and have a genome composed of eight single-stranded negative sense RNA (-ssRNA) segments. The natural reservoirs for influenza A viruses (IAV) are aquatic and migratory birds. However, these zoonotic viruses can also infect domesticated animals such as poultry and swine, as well as humans (**Figure 1**). The zoonotic nature of IAVs, coupled with humans encroaching on animal habitats (1, 2), has increased the likelihood for emerging avian influenza viruses to jump the species barrier and infect humans. As such, these viruses represent a major pandemic threat and vaccine development and pandemic preparedness are a global priority (2).

**Figure 2:**
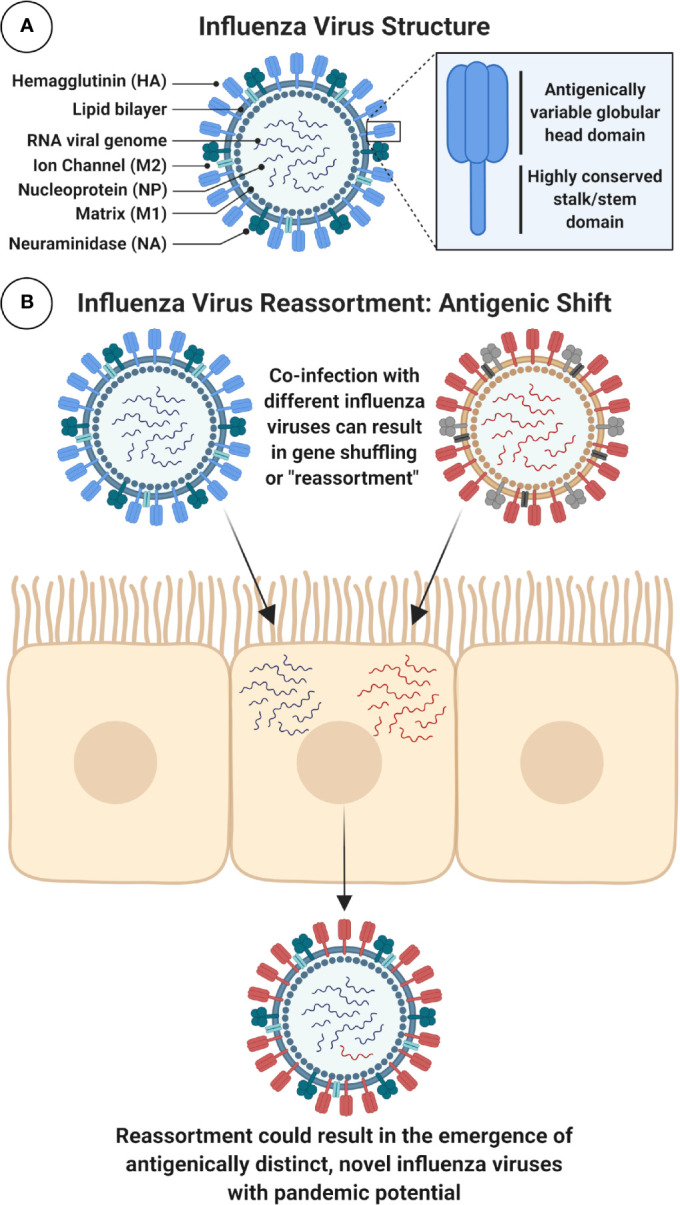
Schematic Diagram of IAV Structure and Reassortment. **(A)** Figure shows a schematic cross-section of the influenza virus virion with main components labeled. Surface glycoproteins, trimeric hemagglutinin (HA) and tetrameric neuraminidase (NA), play important role in viral entry and egress and are major targets for immune responses following infection or immunization. In particular, the highly conserved stalk domain of HA is a target for universal influenza virus vaccines. *Note:* HA stalk and NA stalk are not shown as trimeric or tetrameric structures. Internal, highly conserved antigens matrix protein-1 (M1) and nucleoprotein (NP) are targets for cytotoxic T lymphocytes (CTLs). *Note:* icons for NP, which coats the viral RNA, and the viral ribonucleoproteins (vRNPs) which contain viral RNA, NP and polymerase are not shown. **(B)** Influenza A viruses (IAVs) can evolve to generate viruses with pandemic potential by antigenic shift, using a process of genome reassortment. Co-infection of susceptible cells with more than one distinct IAV can result in the selection of progeny with shuffled gene segments and potentially a new HA or NA, against which humans have no prior immunity. Figure created with ^©^BioRender - Biorender.com.

IAVs are phylogenetically sub-divided according to their surface glycoproteins, the viral hemagglutinin (HA) and neuraminidase (NA) (**Figure 2**). To date, 18 HA and 11 NA subtypes have been identified, although this includes two bat IAV-like HAs (H17, H18) and NAs (N10, N11) (3). Distinct HA subtypes are classified into two groups, group 1 (G1): comprised of H1, H2, H5, H6, H8, H9, H11, H12, H13, H16, and the bat HAs, and group 2 (G2), which includes H3, H4, H7, H10, H14, and H15 HAs (4). The HA protein is immunodominant and is therefore a major target for neutralizing antibodies (NAbs). As a result, it is also the main focus for seasonal influenza virus vaccines. However, IAV viruses evolve and mutate using processes known as *antigenic drift* and *antigenic shift*. Antigenic drift is the accumulation of mutations in the HA (and other proteins) incurred by the error-prone viral RNA-dependent RNA polymerase, often in response to selective pressure from the host. This can result in the evasion of pre-existing NAbs elicited by natural infection or prior vaccination, leading to reduced vaccine effectiveness (5, 6). Alternatively, the segmented nature of the viral genome can result in genome reassortment if more than one IAV simultaneously infects the same cell, creating progeny viruses with a hybrid combination of segments (7). This internal shuffling of genome segments can result in the exchange or incorporation of a novel HA or NA glycoprotein on the virion surface, in a process known as *antigenic shift* (**Figure 2**). This has the potential to result in a novel subtype, for which the human population would be immunologically naïve. Unlike influenza B and C viruses which mainly infect humans and therefore limit this scenario, IAVs can infect many different species including poultry, swine, and other mammals (8). The majority of reassortments result in defective progeny viruses: due to incompatibility between reassorted segments and virus-associated packaging constraints (9), or as a result of species-specific host restrictions which can negatively impact on multiple stages in the virus life cycle (10). For example, a crucial human host protein ANP32A, can confer species-specific restrictions on the avian influenza virus polymerase, limiting the ability of avian viruses to replicate efficiently in human cells (10, 11).

Host receptor tropism determinants can also restrict the occurrence of reassortment. IAV HA proteins bind to host cell sialic acid (SA) receptors, predominantly using SAs attached to galactose with α2,3 linkage (SA α2,3-Gal) or α2,6 linkage (SA α2,6-Gal) (12). Human and classical swine IAVs preferentially bind to α2,6 linked SAs, while avian IAVs preferentially bind to α2,3 linked SAs. SA receptors are mostly found on epithelial cells, with α2,3 linked SAs found in the intestines and respiratory tract of birds (and the lower respiratory tract of humans) (13), while α2,6 linked SAs are mostly found in the respiratory tract of humans and pigs (2, 12, 14–16). Intermediary hosts, such as pigs, play a role in the adaptation of IAVs by acting as a “mixing vessel” and facilitating reassortment, as expression of both α2,3 and α2,6 linked SAs enables them to be simultaneously infected by both human and avian influenza viruses (17) (**Figure 2**). In addition, swine ANP32A has been shown to support replication with the avian virus polymerase (18), further supporting the role for pigs as “mixing vessels” for the emergence of reassortant viruses with pandemic potential (18). The process of antigenic drift can also contribute to the adaptation of avian influenza virus HAs, by facilitating a switch in preference for α2,3 linked SAs to α2,6 linked SAs, or in the viral polymerase (mutation PB2-E627K) (19, 20). If these modifications retained stability and compatibility with other IAV proteins, there is concern that this could facilitate sustained human-to-human spread of avian influenza viruses (21, 22).

## Avian Influenza Viruses

Avian influenza viruses are divided into two main categories on the basis of their pathogenicity in chickens. Highly pathogenic avian influenza (HPAI) viruses cause high mortality in poultry due to their capacity for disseminated, systemic infection (2). This pathogenicity is attributed to the presence of a multi-basic cleavage site within the IAV HA protein. The precursor HA protein, HA0, is cleaved into the HA1 and HA2 subunit, the latter of which is required for membrane fusion and viral entry. HA0 cleavage is normally mediated by trypsin-like proteases for HA0 from human IAVs and low-pathogenicity avian influenza (LPAI) viruses. Trypsin-like proteases are anatomically restricted to the respiratory tract in humans, and the gastrointestinal tract in birds. As such, viral replication following infection with human IAV and LPAI viruses is largely localized to these organs (2). In contrast, the polybasic cleavage site in HPAI viruses, restricted to H5 and H7 subtypes, can be cleaved by proteases which are ubiquitously expressed, facilitating disseminated, extra-pulmonary replication and consequently, severe disease. Although infrequent, sporadic instances of direct bird-to-human transmission of HPAI have occurred. The first report of such a spillover event was recorded in Hong Kong in 1997 following an outbreak of HPAI H5N1 (23, 24). Since 2003, H5N1 viruses have caused a total of 861 laboratory-confirmed cases and 454 deaths. The first report of human infection with HPAI H7N7 was in 2003 in the Netherlands, resulting in 89 confirmed infections and one death (25, 26). In 2013, H7N9 emerged in China and to date has resulted in 1568 laboratory-confirmed cases and 615 deaths (27).

Ducks are mostly migratory birds with α2,3 linked SAs on their intestinal epithelium (12). Studies have shown that several duck species can be infected with, and spread IAVs while showing no clinical signs (28). However, strains of IAV that ducks carry can be highly pathogenic to land fowl, including chickens. The migratory nature of certain water fowl, coupled with the absence of symptoms while carrying IAVs, has been implicated as a major, and unavoidable facilitator for the global spread of IAVs (29). While human contact with wild birds is uncommon, poultry are routinely farmed and present at live animal markets in many countries, which provide an opportunity for human and avian IAVs to co-infect and reassort (**Figure 2**). When farmed poultry are infected by HPAI, containment measures include mass culling, which can have a substantial financial impact. For example, the 2014/2015 H5N2 outbreak in the USA resulted in the death or culling of over 50 million poultry, and was estimated to have a negative economic impact of over $3 billion (30). In low income countries, financial implications can drive smallholder poultry farmers to respond to poultry deaths by rapidly selling stocks, often at markets, which does not help with containment of emerging viruses (31). Despite the obvious benefits of vaccinating poultry in terms of biosecurity, routine vaccination of poultry has cost implications, which means that flock depopulation is a more cost-effective control strategy in many countries (32, 33). It is clear that pandemic preparedness strategies including one-health vaccine development, global surveillance, and data sharing will be crucial in limiting the spread of emerging avian influenza viruses.

## Overview of Adaptive Humoral and Cellular Immune Responses Against Influenza Virus

Ideally, vaccines designed to protect humans against avian influenza viruses should be rapidly customizable and scalable, and should elicit broad, protective immune responses following a single shot. An overview of the types of immune responses which are important in protection from influenza virus infection and disease, and how those responses are measured, are provided below.

## Humoral Immunity

Several methods exist for measuring humoral immunity to influenza virus Ags. ELISA assays are a straightforward and quantitative assay to determine the breadth of, or concentration of antibody (Ab) binding to a range of viral proteins. In addition, they can be used for epitope mapping, which may aid in the identification of new vaccine targets. Adapted ELISAs which measure Ag-specific Ab isotypes or IgG subclasses can also be informative in evaluating the phenotype of response following immunization with different vaccine platforms: including mucosal Abs, or Ab subclasses which have a preference for engaging Fc-mediated effector functions. However, ELISA assays only measure binding-specificity but do not confirm whether Abs are functional and capable of preventing infection.

**Figure 3:**
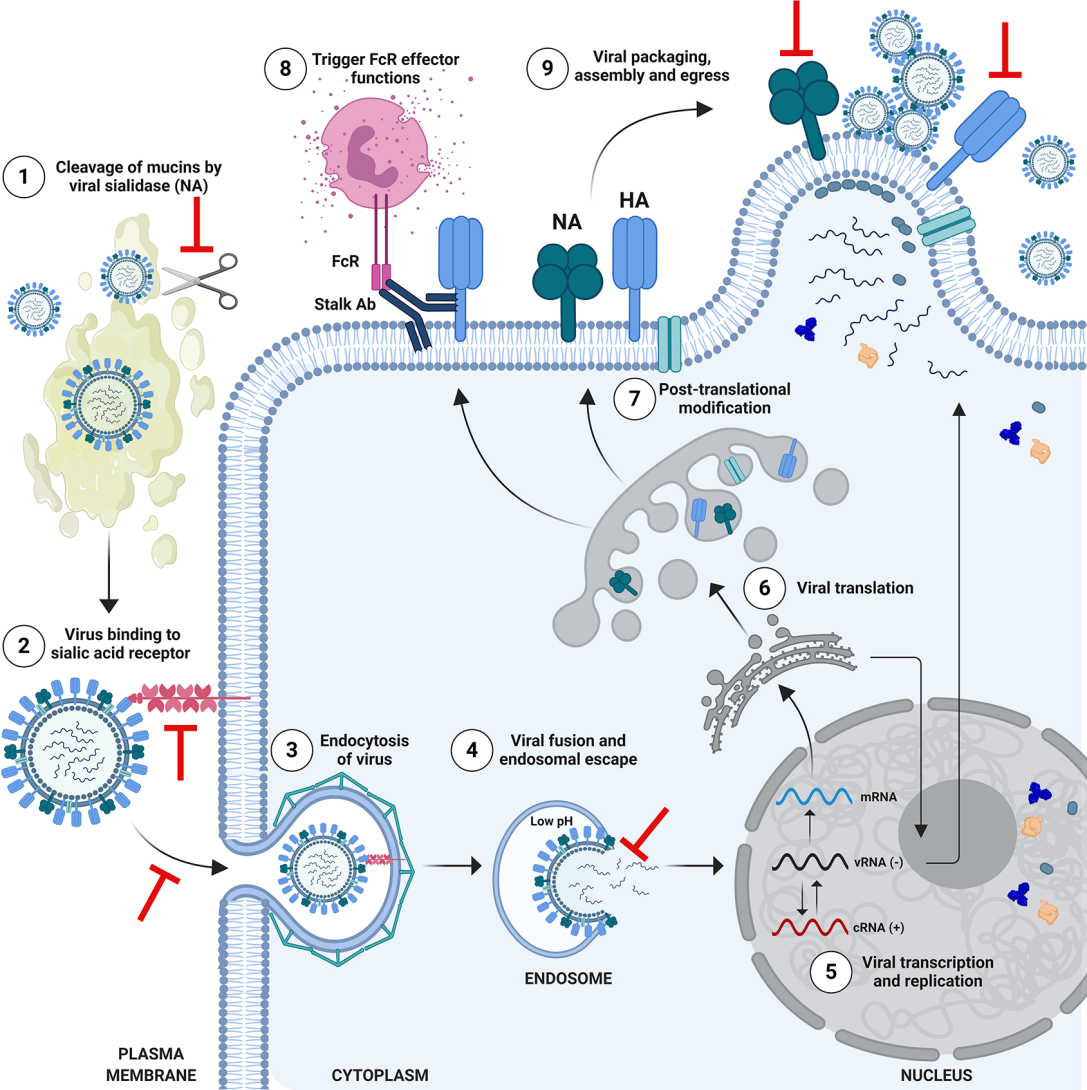
Schematic of Influenza Virus Life Cycle and Targets for Protective Antibodies. The life cycle of influenza viruses has several major steps in which inhibition by neutralizing or protective antibodies can occur. (1) Viral entry in the respiratory tract is facilitated by the enzymatic activity of the viral neuraminidase (NA), which cleaves mucins to allow access to respiratory cells. Anti-NA antibodies, or anti-hemagglutinin (HA) antibodies which block the enzymatic function of NA could potentially inhibit this process. (2) Viral entry is mediated by binding of the head of HA to sialic acid receptors on the surface of cells, followed by endosomal escape by fusion of the viral and endosomal membrane. Antibodies which bind to the HA head domain can block this interaction and can confer sterilizing protection from infection. (3) Alternatively, neutralizing antibodies against HA can block the post-binding internalization of influenza virus, or (4) its’ ability to fuse and escape from the endosome. (5) Viral ribonucleoproteins (vRNPs) are imported into the nucleus for viral transcription and replication. (6) mRNAs exported to the cytoplasm for translation. (7) HA and NA are trafficked to the Golgi for post-translational modification and subsequent presentation on the cell surface. Selected proteins return to the nucleus to participate in viral replication. Progeny vRNPs are exported out of the nucleus towards the plasma membrane for subsequent assembly and virion formation. (8) Anti-HA stalk antibodies can recognize HA on the surface of infected cells and engage Fc-mediated effector functions such as antibody-dependent cellular cytotoxicity, targeting the infected cell for degradation. (9) Viral packaging, assembly and egress takes place at the plasma membrane. This process can also be a target for anti-HA or anti-NA antibodies, which block egress. Anti-NA antibodies can do this by preventing new virions from being released from the surface of infected cells, or by the absence of NA activity causing new virions to aggregate. Figure is adapted from Krammer, 2019 (4). *Note:* icons are not to scale. HA stalk is trimeric (*not shown*) and NA stalk is tetrameric (*not shown*). Figure created with ^©^BioRender - Biorender.com.

Protective and/or NAbs which bind to HA can block viral entry through a range of mechanisms (**Figure 3**). The HA protein is composed of the globular head, and the stalk/stem domain (**Figure 2A**). HA is responsible for entry, facilitated by binding to SA on the cell surface followed by membrane fusion: a process which is mediated following a drop in pH in the endosome, triggering conformational changes in the HA to expose a fusion peptide which fuses the viral envelope with the endosomal membrane. The receptor binding site (RBS) is located in the HA head. Abs recognizing the HA head can confer sterilizing protection by blocking viral entry and thereby preventing infection. NAbs can also recognize the HA stalk domain and can prevent viral entry or egress. In recent years, an important role for stalk-specific, *non-neutralizing* Abs which are broadly cross-reactive has been identified. Many of the latter Abs are non-neutralizing *in vitro* using classical microneutralization (MN) assays. However, it is important to emphasize that this class of Abs are protective *in vivo* and function by engaging Fc-mediated effector functions *in vivo*, such as Ab-dependent cellular cytotoxicity (ADCC) (34, 35) or Ab-dependent cellular phagocytosis (ADCP) (36).

### Hemagglutination Inhibition Assay

The interaction between the HA head and SA receptors is the basis for the hemagglutinin assay (HA), or the hemagglutinin inhibition assay (HAI), which measures Abs that block this interaction. Influenza virus binding to red blood cells (RBCs) causes them to agglutinate and form a lattice. In the HAI assay, sera are first treated with receptor destroying enzyme (RDE) to remove non-specific inhibitors and serial dilutions of sera are pre-incubated with a known quantity of influenza virus, and standard amounts of RBCs subsequently added to the wells. Following an incubation period, the wells are read and the HAI titer identified as the last serum dilution where agglutination was inhibited, as observed by a dense pellet of RBCs in the well. A serum HAI^+^ titer of 1:40 is considered to be a correlate of protection in humans on the basis of a 50% reduction in risk for influenza virus infection (37).

### Microneutralization Assay

MN assays can be carried out as a multi-cycle replication assay (i.e., to measure inhibition of entry and/or egress following addition of Ab prior to, or after viral infection), or as a single-cycle replication assay (to measure inhibition of entry only). The multi-cycle MN assay is performed using a serial dilution of RDE treated sera with TPCK-trypsin, adding a set quantity of virus and pre-incubating before addition of the suspension to cells. Cell supernatants are collected and a HA assay is performed. Alternatively, the single-cycle MN assay is performed in the absence of trypsin and can identify Abs which prevent entry, using an immunostaining-based method as the readout, detecting viral nucleoprotein or HA expression.

### Abs With Fc-Mediated Effector Functions

Several *in vitro* assays exist to measure Fc-mediated effector functions. Mononuclear leukocytes and polymorphonuclear leukocytes can both participate in ADCC, and can be evaluated *via* chromium-release, lactate dehydrogenase-release (38) and esterase-release assays (39), flow cytometry based viability (40) and perforin deposition assays (41). More recently, reporter assays have been developed to measure specific Fcγ-receptor (FcγR) activation. To evaluate the Fc-effector potential of broadly cross-reactive stalk Abs, cell lines expressing different HAs are used. Activation requires a two-contact interaction involving engagement of the Fc portion of the stalk-binding Ab with FcγR on the effector cell, in addition to binding of the HA head to SA on the cell surface (42–45). The ADCC reporter assay has been validated for both human serum and monoclonal Abs (mAbs) against measurement of CD107a cytotoxic degranulation marker on primary NK cells using a FACS-based readout (46). ADCP is typically measured using a cell-based assay, where Abs/serum are pre-mixed with virus to form immune complexes, followed by addition of phagocytes and quantification of internalized virus, which can be achieved *via* RT-PCR, or ELISA-based and fluorescence-based methodologies (36, 47).

## Cellular Immunity

Although T cells cannot confer sterilizing immunity, there is evidence in animal models (48, 49) and humans that they can contribute to limiting disease severity (50), reducing symptomatic infection and viral shedding (51–53). T cells have also been shown to correlate with the NAb response to influenza, with CD4^+^ T cells potentially augmenting the NAb response (54). Considering their capacity for heterosubtypic reactivity, T cells may be the first line of defense and could have an impact at a population level (55) in mitigating the severity of early waves in an emerging avian influenza virus pandemic.

However, widespread implementation of T cell assays is limited by the fact that assays are complicated, require more extensive training, and expensive reagents and equipment. Antigen-presenting cells (APCs) sample exogenous viral Ags or debris from dead/dying cells, and can present epitopes to CD4^+^ T helper cells *via* major histocompatibility complex (MHC) class II. Such helper cells differentiate into different T helper subsets depending on secondary signals (49). Ag-specific CD8^+^ CTLs elicited by prior infection or immunization can recognize influenza-infected cells following presentation of viral peptides on the surface of cells *via* MHC class I (56). Following recognition, these infected cells are subsequently targeted for destruction, limiting viral replication and spread. APCs can also cross-present exogenous influenza Ag to CD8^+^ T cells (57, 58). Popular methods for quantifying Ag-specific T cells are the Enzyme Linked ImmunoSpot (ELISpot) assay and a range of flow cytometry techniques to enumerate and phenotype the T cell response through fluorescently-tagged Ab staining.

### ELISpot Assay

PBMCs can be pulsed with Ag (overlapping influenza peptides, protein or whole virus) to stimulate an existing T cell response to that Ag. The ELISpot is a sandwich ELISA using a capture Ab which binds molecules of interest (eg. cytokines) secreted from T cells undergoing stimulation, followed by use of biotinylated secondary Ab, enzyme-conjugated streptavidin and a development substrate. The readout is based on the formation of visible spots at the location of each responding Ag-specific T cell. Unlike flow cytometry-based T cell assays, ELISpot does not determine whether the responding T cell type is CD4^+^ or CD8^+^, however assay sensitivity is substantially higher (59). ELISpot assays can be modified to measure antigen-specific or total immunoglobulin from B cells, or adapted to use a fluorescent readout, termed a Fluorospot assay, which can detect multiple secreted molecules (60).

### Intracellular Cytokine Staining (ICS) Assay

PBMCs can be stimulated with Ag followed by surface and intracellular staining, to enable the identification of CD4^+^ and CD8^+^ T cells and the cytokines or effector molecules they express (61, 62). Unlike the ELISpot assay that captures secreted effector molecules (eg. IFN-γ), this assay chemically inhibits protein secretion from the Golgi complex, resulting in the intracellular accumulation of upregulated molecules during stimulation. Unlike ELISpot assays, flow cytometry uses a multi-parameter staining readout on a cell-by-cell basis, permitting the analysis of individual cell responses and polyfunctionality.

### MHC Class I/II Multimer Staining

*Ex vivo* MHC multimer staining is a technique by which known epitopes to CD4^+^ or CD8^+^ T cells are targeted through use of specific peptides complexed with MHC (pMHC), and bound in multimeric (ie. tetrameric, pentameric) formation to a fluorescent tag. These multimeric complexes bind the corresponding T cell receptor of cells that recognize the influenza peptide in the pMHC, permitting identification of Ag-specific CD8^+^ T cells using pMHC class I (63), or CD4^+^ T cells using pMHC class II (64). This can be combined with surface staining (e.g., memory markers, CD45RA and CCR7) (65, 66) to provide more comprehensive phenotyping of the Ag-specific cells, without the need to detect a response *via* direct Ag stimulation.

## Beyond Traditional Correlates of Protection

As innovative universal influenza vaccine platforms and approaches are developed, the field needs to move beyond traditional assays to measure correlates of protection. For example, the HAI assay cannot quantify broadly-reactive Abs recognizing the HA stalk. Unlike IIV-based vaccines, many alternative vaccine platforms elicit robust cellular immune responses (67–70). Substantial differences in how assays to measure cellular immune responses are performed makes direct comparisons between pre-clinical and clinical studies challenging (71). This extends to differences in the specific Ags being evaluated, as well as differences in the cell number, stimulating peptide concentration used, all factors which can affect the results. Furthermore, the identification of a particular phenotype of cellular immune response does not confirm a role in protection. Therefore, much information remains to be learned from well-designed longitudinal cohort studies of natural infection, and human challenge studies (72). The licensure of new vaccine candidates will be dependent on the implementation and standardization of a broader range of assays to identify and measure correlates of protection (72).

## Approaches for Avian Influenza Virus Vaccines

**Figure 4:**
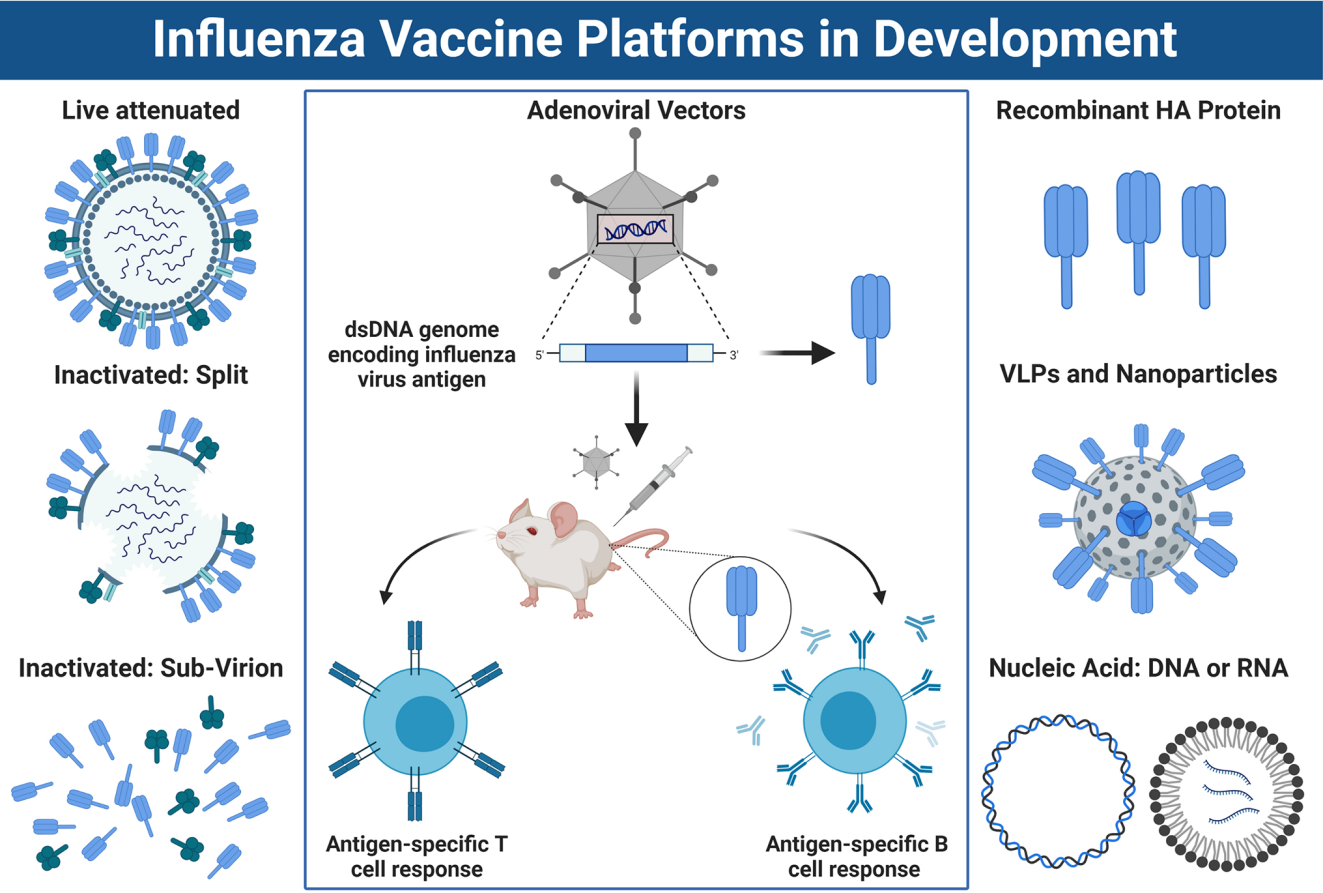
Approaches for Influenza Vaccine Development. *Left panel*: A schematic overview of conventional influenza virus vaccine platforms, including the live attenuated vaccine (LAIV), the split virion inactivated influenza vaccine (IIV) or IIV sub-virion vaccine, which has HA>NA content. *Right panel:* Newer vaccines being developed include recombinant HA protein, virus-like-particles or nucleic acid-based vaccines such as DNA or mRNA platforms. *Center panel:* Schematic overview of how non-replicating adenoviral (Ad) vectored vaccines work. DNA sequence encoding an influenza virus antigen is inserted into the dsDNA genome of the Ad vector under the control of a powerful promoter to drive expression. Once immunized, the DNA sequence coding for the influenza antigen is transcribed into mRNA and translated into protein which is expressed inside the host cells at the site of injection and/or within draining lymph nodes. This results in a robust CD8^+^ T cell response, as well as humoral immune responses directed towards the encoded transgene antigen. *Note:* the trimeric stalk of HA, or tetrameric stalk of NA are not shown in the diagram and icons are not to scale. Figure created with ^©^BioRender - Biorender.com.

HPAI viruses represent an ongoing pandemic threat, and unfortunately, it is difficult to predict which subtype will spillover and cause the next epidemic or pandemic. As a result, there is significant interest in developing vaccines which provide broad protection from a range of emerging IAVs. Conventional vaccine platforms used to protect against influenza virus, such as IIV or LAIV, predominantly rely on production in embryonated chicken eggs. However, many novel vaccine candidates are under development which do not rely on egg-based production (**Figure 4**).

### IIV

Inactivated vaccines can comprise of several formulations including whole inactivated virus vaccines (WIV), split-virion or sub-unit vaccines, each with their advantages and disadvantages. WIV vaccines are generally chemically inactivated and are robustly immunogenic, thought to be due to their crude preparation and subsequent stimulation of innate immune signaling pathways by residual viral RNA (73). Depending on the method of inactivation used, WIV vaccines can retain the structural integrity or functional activity of the HA and NA, the two major targets for NAbs (4). Furthermore, as WIV vaccines retain internal Ags, these may also facilitate boosting of cross-reactive T cell responses to conserved viral proteins such as nucleoprotein (NP). However, WIV vaccines have fallen out of use in recent years due to their increased reactogenicity relative to more highly-purified formulations. Split-virion or sub-unit vaccines represent WIV vaccines which have undergone additional treatment with detergents to further purify virions into membrane components bearing both HA and NA (split-virion), or almost purely HA-based immunogens (sub-unit) (4). As a result of manufacturing processes which enrich for HA content, immune responses to the latter vaccines are almost exclusively skewed towards HA (74). Unfortunately, the use of IIV-based vaccines for avian influenza viruses with pandemic potential has been hampered by poor or variable immunogenicity requiring high Ag doses (75), multiple immunizations (75, 76) or the inclusion of adjuvants to achieve levels of Abs which would be considered protective (76–79).

### LAIV

LAIV platforms are cold-adapted and are designed to be administered to the upper respiratory tract (URT) *via* intranasal (*i.n.*) immunization. Cold-adaptation allows the LAIV to undergo limited replication in the cooler environment of the URT, but does not facilitate dissemination to the lung. The aim of this vaccine is to stimulate a multi-faceted response, with mucosal immunity in addition to priming/boosting of cellular immunity (80–82). IIV and LAIV vaccine formulations are similar in that they aim to stimulate protective Abs directed towards HA, and to a lesser extent, NA. Although there is added potential to elicit cross-reactive immunity with LAIV as compared with IIV (82), there are safety concerns regarding the use of LAIV vaccines with avian HAs, as it could be argued that immunization might facilitate reassortment if the recipient became simultaneously infected with a circulating seasonal IAV (**Figure 2**). In addition, LAIV vaccines are not suitable for use in all populations (i.e., pregnant women, immunocompromised individuals).

However, sub-optimal immunogenicity and safety concerns are not the only challenges: employing conventional IIV/LAIV platforms in the development of avian influenza vaccines also presents several unique manufacturing hurdles. A major issue is the long manufacturing time, which is not conducive to rapid responsiveness in an emerging pandemic scenario. Strain selection usually takes place 7–8 months prior to influenza season (83), and production can be achieved within 5–6 months in the best-case scenario: when suitable seed stocks are identified and recommended by WHO on time, and when these viruses grow to sufficient titers. However, under unexpected circumstances the process can become protracted, with production ranging from 6–8 months (2, 84–86). Another issue is the over-reliance on production in embryonated eggs and the potential for significant reductions in supply should a HPAI epidemic result in decimation of poultry, and subsequently eggs needed for manufacturing. In parallel with this is the fact that HPAI viruses and derived vaccine seed stocks can be embryo-lethal, leading to challenges in propagating viruses in eggs to make vaccine stocks. Additionally, handling HPAI viruses intended for vaccine development in enhanced BSL-3 biocontainment facilities requires specialized staff and procedures which increases costs. To overcome this, non-pathogenic surrogate avian viruses can be used, or HPAI viruses can be genetically modified using reverse genetics (2). However, production would benefit from alternative platforms which are safe, easily adaptable, elicit robust and broad protective immunity and possess manufacturing characteristics which are compatible with stockpiling and pandemic preparedness.

Newer vaccines to the market, such as recombinant HA (rHA) produced in insect cells (i.e., Flublok^®^) or IIV vaccines grown in mammalian cells (i.e., Flucelvax^®^) could overcome the dependency on egg-based manufacturing and the protracted manufacturing process. Recombinant protein-based vaccines could certainly be scaled up more rapidly in response to an emerging pandemic. However, in the context of avian influenza vaccines, both rHA and IIV-based platforms may still be affected by inherently poor immunogenicity, in addition to the fact that these particular vaccines are limited in their ability to stimulate robust cellular immunity. Therefore, when designing vaccines to protect against HPAI viruses, we should consider platforms which can elicit immune responses with increased breadth, or which simulate both arms of the adaptive immune response to several antigen targets simultaneously, rather than over rely on HA as the sole target.

## Novel Vaccination Strategies to Increase Influenza Vaccine Breadth

The development of a universal influenza virus vaccine has become a significant research priority in recent years. Several position papers have outlined major gaps in our knowledge and have highlighted the need to invest in innovative approaches to achieve this (87–89). One strategy is to expand the repertoire of vaccine platforms under investigation, which might help to overcome the reliance on egg-based manufacturing, as well as increase vaccine breadth and durability. Alternatively, as different vaccine delivery vehicles elicit a differential phenotype of immunity, distinct platforms could be used as tools to better understand which components of the immune response are desirable for broad efficacy, and could help to identify new correlates of protection. Although beyond the scope of this review, diverse vaccine platforms including conserved peptides (90), bivalent peptide conjugate vaccines (i.e., NCT00851266), DNA (91, 92), mRNA (70, 93), nanoparticle (94, 95), or virus-like-particle (VLP) (96, 97) based vaccines are undergoing evaluation as universal influenza virus vaccines, which could also protect against emerging pandemic HPAIs. Many of these alternative platforms are attractive because they can facilitate the delivery of multiple conserved Ags/epitopes simultaneously, some have inherent immunostimulatory or innate “adjuvanting” qualities which could increase breadth, some platforms present Ag in novel conformations such as repetitive particulate formulations (i.e., nanoparticle or VLP), and others are amenable to rapid customization and pandemic responsive scale-up. In particular, viral vectored vaccines fulfil many of these criteria. Their ability to enter cells and deliver their nucleic acid genome allows them to trigger immunostimulatory pathways, which can create an environment which enables increases in immunological potency once the transgene is expressed (98).

## Non-Replicating Adenoviral Vectored Vaccines

Non-replicating Ad vectored vaccines are an attractive platform for vaccine development (**Figure 4**). They have a stable dsDNA genome, they can be rendered replication-incompetent (non-replicating) by deletion of the E1 region which is essential for viral replication, they can tolerate the insertion of large heterologous transgene Ags and promoters driving their expression (up to 7.5 kbp), and a number of vectors are available for vectorization (98). More importantly, they have a strong track record of use in human clinical trials (99, 100) and are well-established to be safe and immunogenic when used as vaccines for major infectious diseases in young infants (101–103), healthy adults (67, 68, 104–107), older adults (67, 68) and even immunocompromised individuals (108). In recent months, their suitability for rapid, pandemic responsiveness has been exemplified by the fact that several Ad vaccine platforms (i.e., Ad5 (109, 110), Ad26 (110, 111), and ChAdOx1 (112, 113)) have advanced through pre-clinical studies in mice (114), hamsters (115), pigs (114), and non-human primates (NHP) (111, 112), and are now leading the way in clinical trials for the newly emerged coronavirus, SARS-CoV-2 (i.e., NCT04324606, NCT04313127, NCT04436276, NCT04436471, and NCT04437875) (109, 110, 113).

Depending on the particular Ad serotype selected as a vaccine, Ad vectors elicit potent cellular immunity (largely CD8^+^) (116), in addition to humoral immunity directed towards the encoded transgene Ag. This is due in part to their ability to stimulate multiple innate immune signaling pathways upon viral entry (117–121), as well as their capacity for persistent transgene expression *in vivo* (98, 122). Adenoviruses are classified into species groups A–G, with Ad vectors derived from species groups C, D, and E exhibiting the highest immunological potency when used as vaccines (98, 116). The most commonly used “prototype” Ad vector is human adenovirus type-5 (HAdV-C5, *referred to as Ad5 throughout this review*), a potently immunogenic vaccine which unfortunately has high seroprevalence in humans, possibly limiting its potential for broad clinical applications (98). Issues associated with pre-existing immunity in humans has driven scientists to vectorize a range of alternative, rare serotype human Ad vectors, or Ad vectors derived from NHPs, great apes and other animal species. Novel Ad platforms which have been evaluated in pre-clinical models as vaccines against avian influenza virus include species C vectors HAdV-C5, HAdV-C6 (123), species D vectors HAdV-D26 (124), HAdV-D28 (124) and HAdV-D48 (124), species E human virus HAdV-E4 (125, 126), along with species E viruses isolated from chimpanzees ChAdV-7 (ChAd7) (127), ChAdV-68 (AdC68) (128), and ChAdOx1 (129–131). Other novel vaccines include the use of porcine vector PAdV-3 (132), or bovine Ad vector BAdV-3 (133). Aside from the contribution of the specific Ag selected for incorporation into an Ad vaccine to overall immunogenicity or efficacy (*discussed in more detail below*), the relative immunological potency of the chosen Ad vector platform can vary significantly. It is considered that a combination of factors contribute to the hierarchy of immunogenicity when comparatively evaluating distinct Ad vaccines (98). These include the persistence of transgene expression *in vivo*, and subsequently, the magnitude of the ensuing immune response (122, 134, 135), as well as the preferential induction of key innate immune signaling pathways—combined with the avoidance of Type I IFN stimulation at early time-points post-immunization (134, 135). Additional factors such as the cellular tropism or receptor usage of the selected Ad vector, the route of vaccine administration and dose, can also play a role in modulating the inherent immunogenicity of different Ad vaccines (98). These concepts are the subject of a comprehensive review article recently published by our group (98).

## Antigen Targets for Avian Influenza Vaccine Development

The high mutability of the HA, a result of antigenic drift, ensures that conventional vaccine platforms (i.e., IIV) elicit largely strain-specific humoral immunity. A vaccine based on this premise would provide little or no protection against antigenically diverse avian influenza viruses, particularly if we consider the unpredictable nature of cross-species transmission events by these zoonotic viruses. Therefore, it is difficult to rely on current licensed vaccine platforms for pandemic preparedness against HPAI. Ideally, we need novel vaccines which are capable of stimulating broad, heterosubtypic immunity against a wide range of avian influenza viruses, in addition to developing platforms which are amenable to rapid production and scale-up, or suitable for stockpiling. One way to achieve increased breadth of protection from a vaccine is to select viral Ags which are highly conserved as targets. Such Ags usually play crucial functional or structural roles in viral replication or assembly, making them unable to tolerate significant mutations without compromising viral fitness. Several key targets which are currently under investigation for universal influenza virus vaccine design are discussed below.

### HA

**Figure 5:**
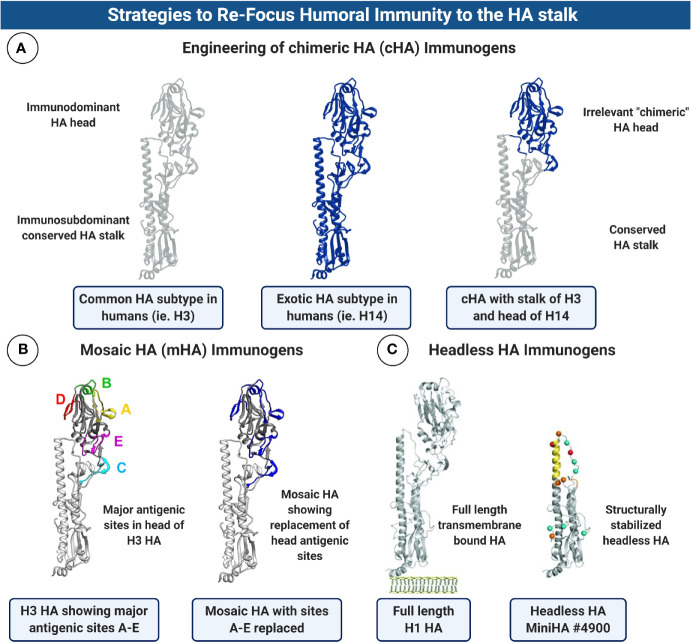
Strategies to Re-focus Humoral Immunity to the HA stalk. **(A)** Schematic diagram showing the substitution of the HA head domain to make chimeric HA (cHA) immunogens. The concept behind this approach is to graft an exotic HA head, for which humans have no prior immunity, to the stalk of a HA subtype which is common in humans (ie. H1 or H3). Sequential immunization with cHA immunogens in which the exotic head is swapped with each boost can re-focus humoral immunity to the conserved HA stalk. *Note:* Structures are schematic and do not represent authentic junctions for substitution of the HA head region. **(B)** Mosaic HA (mHA) design is conceptually similar to cHAs but only the major antigenic sites in the HA head domain are swapped for comparable regions in an exotic HA. This can be used as an alternative approach to re-focus antibodies towards the HA stalk domain, with the added benefit of retaining possible conserved epitopes in the HA head. mHA structures kindly provided by Dr. Felix Broecker and Prof. Peter Palese, ISMMS. **(C)** Structurally stabilized headless HAs have been engineered which completely lack the immunodominant HA head domain, allowing boosting of immune responses towards the stalk only. HA structures in **(C)** are reproduced with permission from Impagliazzo et al. (147). Reprinted with permission from AAAS (License 4907650635299). Figure created with ^©^BioRender - Biorender.com.

HA is the most abundant glycoprotein on the surface of the influenza virion. Although HA is subject to antigenic variation which can negatively impact on vaccine effectiveness, it does possess a highly conserved domain which is an ideal target for universal influenza virus vaccines. HA is composed of two main structural domains, the *antigenically variable* and *immunodominant* HA head domain, and the *highly conserved*, but *immunosubdominant* HA stalk/stem domain (**Figure 2A**). As previously stated, conventional vaccines elicit largely strain-specific humoral immune responses predominantly focused on the antigenically variable HA head domain. The *immunosubdominance*, or poor immunogenicity of the HA stalk in this context is well documented (i.e., head > stalk) (136). However, advances in innovative HA immunogen design in recent years has enabled re-focusing of humoral immune responses towards this *immunosubdominant* HA stalk domain. A major step forward in facilitating the induction of robust stalk-specific immune responses was the development of chimeric HA (cHA) immunogens (137–141), in which the head domain of an exotic IAV HA is grafted onto the stalk domain of a common human HA, the use of mosaic HAs (mHAs), in which the major antigenic sites in the HA head domain have been silenced (142, 143), or the design of structurally stabilized headless HA immunogens (93, 94, 144–148) (**Figure 5**). Alternatively, hyper-glycosylation of the HA head domain through the introduction of N-linked glycosylation sites, can also re-focus humoral immunity away from the head and towards the stalk (149, 150). Although disassociating the HA head from the HA stalk domain, through the use of headless HA-based immunogens, appears to improve the inherent immunogenicity of the stalk (151), sequential immunization approaches are still required to achieve broad protection. In addition, differences in the immunogenicity of the HA stalk as an immunogen when presented in different formulations exist. For example, use of recombinant protein-based HA stalk in a single shot is poorly immunogenic, although this can vary depending on the specific stalk construct used, its associated stability and structural integrity. The immunogenicity of the HA stalk can be improved by use of adjuvants, or by modification of HA stalk constructs through covalent coupling to immunogenic carrier proteins (152) or nanoparticles (94). Although the immunosubdominance of the HA stalk can likely be overcome with the right immunogen and vaccination regimen, the immunological factors which contribute to its subdominance are intriguing. Poor accessibility of stalk epitopes or steric hindrance imposed by the HA head (153), polyreactivity (154) and potentially counterselection of HA stalk Abs with low affinity for B cell receptors (155) and the paucity of MHC II epitopes in the stalk relative to the head which could affect Tfh responses (152), are all mechanisms which have been proposed as underlying factors. Regardless, in support of its potential as a universal vaccine target, numerous studies have demonstrated that sequential immunization with stalk-focused immunogens can confer heterosubtypic protection from lethal challenge in animals (94, 139, 140, 145, 147, 156). For example, sequential immunization with a headless or cHA immunogen with the H1 stalk, can confer protection against a distinct IAV subtype from the same phylogenetic group (i.e., G1 avian influenza H5N1). Importantly, cHA based vaccines have recently been evaluated in clinical trials and have shown that they can effectively boost stalk-reactive Abs capable of recognizing distinct G1 HAs including H1, H2, H9 and H18 in humans (157). In addition, headless HA vaccine candidates are also undergoing clinical evaluation (i.e., NCT03814720).

A major advance in the field was the discovery that broadly cross-reactive HA-stalk Abs can protect using a range of mechanisms which are independent of HAI activity (**Figure 3**). Anti-stalk Abs can be neutralizing, inhibiting the fusion activity of HA by preventing its structural rearrangement and exposure of the fusion peptide, thereby blocking viral entry. HA stalk Abs can also interfere with the enzymatic activity of the viral NA by steric hindrance, blocking viral egress (158). However, more recently we have begun to understand the contribution of *non-neutralizing*, but broadly cross-reactive stalk Abs in mediating protection *in vivo* (72). As the latter class of protective Ab does not always neutralize *in vitro* when using classical assays, such as HAI or MN, their importance was long under-appreciated. We now know that a large number of stalk-reactive Abs can protect *in vivo* by engaging FcγRs (159), and triggering Fc-mediated effector functions such as ADCC (34, 35) or ADCP (36). This is a very important consideration in the evaluation of novel vaccines designed to elicit heterosubtypic immunity, as the current correlates of protection for the licensure of influenza virus vaccines are based on the HAI assay, and stalk-reactive Abs with substantial breadth are HAI^-^. To date, vaccines designed to elicit HA-stalk mediated protection have employed conventional IIV, LAIV or recombinant protein-based platforms, with or without adjuvants (138–140, 156). However, alternative approaches have used nanoparticles or VLPs bearing headless HA. Importantly, all of the innovative HA designs described above (i.e., headless, cHA or mHA expression cassettes) are well-suited for genetic incorporation into non-replicating Ad vaccines (**Figures 4**, **5**).

Some concerns regarding the use of HA stalk as a vaccine target are based on selected studies which have implicated stalk Ab responses in leading to vaccine-associated enhanced respiratory disease (VAERD). A study by Khurana and colleagues reported that pigs immunized with adjuvanted whole-inactivated influenza (WIV) vaccine based on H1, developed enhanced disease following viral challenge with an antigenically mismatched H1N1 virus. The authors attributed this to non-neutralizing stalk Abs which promoted virus membrane fusion activity (160). However, subsequent studies compared the adjuvant used in the latter study head-to-head with WIV immunization using different adjuvants and did not observe VAERD, suggesting that the immune-enhancement effects in the Khurana study were associated with that particular choice of adjuvant (161). Importantly, Braucher and colleagues compared adjuvanted-WIV with an Ad5-based vaccine encoding HA in pigs and showed that unlike WIV, the Ad vector did not induce VAERD, and elicited superior protection against heterologous challenge viruses (162). Antibody-mediated immune enhancement upon challenge with H3N2 viruses in mice has also previously been reported for mAbs which bind to the HA head, or base of the HA head (163). The mechanism was proposed to be destabilization of the HA stalk, resulting in increased viral fusion kinetics. Although experimental studies with epitope-specific mAbs are useful in better understanding mechanisms of protection or disease enhancement, information which will guide next-generation vaccine design, the physiological response to immunization results in a pool of Abs which recognize multiple epitopes, and/or multiple viral antigens present in the vaccine formulation. It is also important to note that stalk Abs are prevalent in humans, boosting of stalk Abs can occur in humans following immunization (164, 165) or natural infection (166, 167), and stalk Abs have recently been identified as a correlate of protection in a household cohort study of natural influenza virus infection (167).

### NA

In addition to HA, the other major surface glycoprotein is the viral sialidase, NA. NA is responsible for cleaving SA from the surface of host cells and plays a role in viral entry (by facilitating movement through mucus in the respiratory tract) (168, 169), in allowing the release of budding virions from the surface of infected cells, as well as preventing the aggregation of released viruses (169–171) (**Figure 3**). In recent years NA has gained interest as a new universal vaccine target (171, 172). Although Abs to NA do not provide sterilizing immunity, they can limit disease severity in animals (173–175), and have been shown to reduce viral release/shedding and symptomatic infection in human challenge studies (176–178). Unlike HA, breadth of reactivity to NA is usually across a particular subtype (i.e., N1) rather than between different NA subtypes (172). Unfortunately, seasonal vaccines do not contain standardized amounts of NA, its stability and abundance in these formulations is low relative to HA. In addition, issues related to antigenic competition between intravirionic HA (dominant) and NA (subdominant), when presented together on the same vaccine platform (i.e., IIV), can preclude the development of robust immunity to NA (179). Therefore, vaccines such as non-replicating Ad vectors, which are capable of driving high-level *in vivo* expression of NA under the control of exogenous promoters, might enable improved immune responses to NA (180). In addition, the *in vivo* expression of NA following immunization using a viral vectored vaccine could overcome issues related to the poor shelf-life stability of NA in current vaccine formulations.

### NP

Influenza virus nucleoprotein (NP) is a structural protein which coats the viral RNA genome, forming the viral ribonucleoprotein complex (vRNP). Although NP has been implicated in mediating the switch from transcription to viral genome replication (181), recent data suggests that NP does not regulate this process (182). Nonetheless, NP is known to interact with other viral proteins, including components of the polymerase complex and M1 (183). With respect to its potential role in conferring heterosubtypic immunity, the high sequence conservation of NP (> 90%) (184) and its role in providing partial protection from influenza virus infection in mice (185), suggests it should be included in next-generation vaccines. In further support of this, NP-specific T cells have been correlated with limiting symptomatic infection and reducing shedding in humans during natural infection and influenza virus challenge studies (51–53), and NP-specific CTLs have been shown to cross-react with avian viruses (82, 186). Moreover, clinical studies have already shown that NP-specific T cell responses can be boosted in healthy adults and in the elderly when using Ad-based vaccines expressing NP as the transgene Ag (67, 68). Although CTL responses to NP cannot provide sterilizing immunity, the inclusion of NP in vaccines designed to protect against emerging avian viruses could help limit disease severity, or virus shedding and replication, both important considerations in mitigating the early impact of a pandemic.

In addition to eliciting T cell responses, Abs to NP have been reported to display ADCC activity (187, 188). Despite NP being an internal virus protein, it can be expressed on the surface of infected cells (189), providing an explanation for its role in triggering Fc-mediated effector functions. In addition, studies have described that human Abs to NP, elicited in response to seasonal influenza viruses, can cross-react with avian influenza strains from both phylogenetic groups, H5N1 and H7N9, and trigger ADCC (188). However, the contribution of NP Abs to heterosubtypic protection from influenza virus challenge in animals is not conclusive, with some reports of protection (190–192), and others describing minimal protection, depending on the challenge virus tested (93). This may be due in part to differences in the IgG subclass profile of the Ab response elicited by different vaccine platforms or in response to influenza virus infection. It is well-established that specific IgG subclasses have differential affinities for activating FcγRs, and as a result, this can have a different outcome on the induction of ADCC (193, 194). Therefore, NP may not be ideal as the primary Ag target but would be well-suited to vaccine platforms which can encode or express more than one Ag simultaneously.

### M1

Matrix protein-1 is an internal Ag which forms a stabilized shell under the IAV envelope (**Figure 2**). M1 therefore plays an important structural role, engaging in interactions with the vRNP (195) and recruiting other viral components which facilitate virion assembly. In addition, M1 has been reported to interact with the cytoplasmic tails of both HA and NA (196, 197). Similar to NP, M1 exhibits a high degree of amino acid sequence identity (> 95%) amongst global IAV isolates (198), also making it an attractive target to elicit heterosubtypic cellular immune responses. Evidence in animal models suggests that M1 responses can confer a degree of protection (192). A dominant HLA-restricted epitope does exist in M1, and individuals with this high-population-frequency haplotype (HLA-A*02) have detectable M1-specific CD8^+^ T cells (199, 200). Clinical trials using a heterologous prime:boost immunization regimen with a chimpanzee Ad vector and MVA expressing NP+M1 as a fusion Ag, demonstrated that although NP T cell responses were boosted significantly following vaccination, M1 boosting was minimal (68). However, in a previous study, volunteers who were HLA-A*02 (7/15) exhibited pronounced fold changes in T cell responses to this M1 epitope (67). Interestingly, modified vaccinia Ankara (MVA) vaccines expressing NP or M1 conferred protection from influenza virus challenge in HLA-A2 transgenic mice (201). However, a role for immune responses to M1 as a potential correlate of protection in humans is currently unclear.

### M2

The M2 protein is an ion channel which is displayed on the virion surface in low abundance, although its density is increased on the surface of infected cells (202). It is involved in virus uncoating during entry, and in the formation of new virions and budding. The ectodomain of M2 (M2e) is highly conserved between all IAVs (203, 204), making it a good target for vaccines (205). Studies in animals first demonstrated that a monoclonal antibody against M2 could protect mice from challenge with influenza virus (206). Subsequent experiments using multimerized M2e fused to hepatitis B virus core (HBc) demonstrated significant protection in mice, which was Ab-mediated (204). In addition, other approaches have tested M2e presented on VLPs (207), or have supplemented conventional IIV-based vaccines with M2e presented on VLPs (208). It is considered that M2e induces protection in a manner similar to broadly reactive stalk-Abs, through engagement of Fc-mediated effector functions, with a crucial role for alveolar macrophages (36, 209). Some disadvantages of using M2e as a universal immunogen include its low abundance on the virus, its small size and therefore restricted space for T cell epitopes, and the fact that despite its high sequence conservation, several phylogenetic lineages of M2e exist (203). Clinical trials based on M2e-fusion proteins with HBc formulations, such as ACAM FLU-A have been registered (NCT00819013 and NCT03789539) (204, 210). Despite reported safety and seroconversion for ACAM FLU-A, no further development of this vaccine has been reported. M2e-based vaccines have also advanced to clinical trials where the vaccine is formulated as a tandem immunogen linked to bacterial flagellin, a TLR5 ligand (NCT00603811) (211). Early studies using low doses of the latter immunogen (0.3 and 1.0 μg) were well-tolerated, and immunogenic, particularly following a second dose. However, higher doses (3.0 and 10.0 μg) of flagellin-M2e were associated with adverse events and reactogenicity. Recently, the use of a full length mutant M2 encoded within an mRNA-based vaccine, displayed promising efficacy against a range of influenza virus challenges, suggesting that alternative platforms for delivery of M2 may be a worthy pursuit (93).

### PB

The viral polymerase complex of IAVs is composed of three subunits: PB1, PB2, and PA. Although not a major focus for universal influenza virus vaccine development, components of the polymerase, namely PB1, may be of interest for vaccine development, particularly from the standpoint of eliciting heterosubtypic CTLs. One study using non-replicating Ad vectors determined that PB1 was not as immunogenic as NP, but this could be overcome using a molecular adjuvanting approach in which PB1 is fused to the murine invariant chain (Ii), increasing its presentation (212). This genetic fusion strategy was originally designed to exploit the canonical role of Ii in MHC II presentation, with a view to augmenting Ag presentation to CD4^+^ T cells, but unexpectedly led to increases in Ag-specific CD8^+^ T cell responses (213, 214). However, despite improved T cell responses, fusion of PB1 to Ii did not translate into robust vaccine efficacy and protection from influenza virus challenge in mice. This is in agreement with another study which determined that PB1 was not effective as a sole Ag in providing protection from challenge (192). What is interesting is the recent implication that CD8^+^ T cell responses to PB1 in humans exhibit unprecedented breadth (215). In particular, two conserved epitopes in PB1 were identified which are restricted by common HLA types, suggesting that the development of novel vaccines which are capable of boosting these responses could elicit broad cross-reactivity in a significant proportion of the human population (215). CD8^+^ T cell responses to one of these epitopes cross-reacted against IAV, influenza B virus (IBV) and influenza C virus (ICV). Therefore, it may be that authentic evaluation of the contribution of PB1 to protection is complicated by differences between studies in mice and humans. However, as stated previously, it is also important to note that the presence of cross-reactive CTLs does not guarantee that they will contribute to increased viral clearance or protection. The phenotype, functional activity and protective capacity of PB1-specific T cells in humans remains to be investigated in more detail.

## Adenoviral Vectors in Development as Vaccines for Avian Influenza

The unexpected pandemic caused by the introduction of SARS-CoV-2 in late 2019, highlighted the importance of accelerating the development of vaccine platforms which have the capacity for rapid scale-up, which already have a strong track-record for use in clinical trials and ideally, which would elicit broad protective immunity against antigenically distinct avian influenza viruses. The suitability of Ad vaccines for many aspects of this endeavor has ensured that they are now center-stage in this global effort, with Ad5, Ad26 and ChAdOx1 vaccines for SARS-CoV-2 already in clinical trials in humans (ie. NCT04324606, NCT04313127, NCT04436276, NCT04436471 and NCT04437875) (109, 110, 113).

Adenoviruses are dsDNA viruses which have three main structural proteins that play a role in their tropism and type-specificity: these include the fiber, which is involved in binding to receptors on the cell surface, the penton base which induces viral internalization and the hexon, the most abundant viral protein in the capsid (56, 98, 216). The flexibility of the Ad platform has facilitated its combination with many innovations in Ag design: such as the ability to encode multiple transgenes, computationally designed consensus Ags (125, 217) and the insertion of antigenic epitopes into the capsid of the Ad vector (218). We already outlined the main Ag targets for achieving broad immunity against influenza viruses. We will now summarize the status of Ad-based vaccines for avian influenza viruses from pre-clinical models, and vaccines which have already entered clinical trials in humans.

### Pre-Clinical

#### Ad5 Vaccines

Several studies have reported the pre-clinical evaluation of Ad5-based vaccines against avian influenza viruses in mouse models (all studies described are in mice unless otherwise stated). In 2006, Hoelscher et al., described the successful construction of an Ad vaccine encoding H5 A/Hong Kong/156/197) which provided protection against antigenically distinct H5N1 influenza viruses (219). Hassan and colleagues described the construction of vaccines encoding full length sequences for H5 (H5N1: A/Vietnam/1203/2004), H7 (H7N7: A/Netherlands/219/2003) or H9 (H9N2: A/chicken/Hong Kong/G9/1997) (220). Interestingly, the authors also constructed a multi-epitope based vaccine (Ad-ME) encoding highly conserved domains, or regions from diverse viral proteins from H5N1: M2e, the fusion peptide of the HA stalk, an immunodominant T cell epitope in NP and the α-helix domain of the HA stalk (another highly conserved target on HA) (221). This vaccine elicited ELISA Ab responses to M2e and the HA fusion domain, but not to the α-helix. In addition, T cell responses to NP were detected by ELISpot. Challenge experiments were set up to test heterologous H5N2 virus, a distinct G1 challenge virus H9N2 and a G2 virus, H7N9. Although viral lung titers were reduced somewhat for the Ad-ME vector upon challenge, these were still significantly higher than the matched HA control vaccines, suggesting that protection was only partial and the inclusion of structurally intact HA immunogens may be required to confer adequate Ab-mediated protection.

With this in mind, many investigators have designed Ad vaccines which express conformationally intact, full length HAs. An advance on this is computationally designed, centralized consensus HA sequences which have now also been applied in the development of Ad-based vaccines, with the aim of increasing the breadth of reactivity against diverse HAs (217). The concept behind this approach is that a computationally optimized broadly reactive antigen (COBRA) (222) sequence would represent the central node of a HA phylogenetic tree. In a study by Webby and Weaver, the authors engineered Ad5- and rare species Ad4 vectors encoding consensus H1 (H1-Con), H3 (H3-Con) and H5 (H5-Con) transgenes and tested them in a heterologous prime:boost regimen in mice (125). Vaccines encoding avian H5-Con induced HAI^+^ Abs with titers ≥40 against two out of three H5 viruses tested. This prime:boost regimen conferred protection from mortality following challenge with divergent H5 viruses, but protection from morbidity (weight loss) was only observed following challenge with the H5N1 A/Vietnam/1203/04 virus, not two other strains.

In an effort to re-focus humoral immunity away from the HA head and towards the conserved HA stalk, Lin et al., designed Ad vaccines encoding H5 (A/Thailand/1(KAN-1)/2004) in which the immunodominant antigenic sites in the HA head were shielded from immune recognition by hyperglycosylation, *via* the introduction of specific glycosylation sites (223). Using a heterologous Ad prime:recombinant protein boost (both with hyperglycosylated H5), the authors demonstrated that these “masked” HAs elicited Abs with greater cross-clade HAI^+^/NAb and anti-RBS ELISA Abs against diverse H5 viruses, as well as inducing Abs to the conserved HA stalk domain (223).

Another strategy to increase the breadth of protection is to target more than one Ag, or use co-administration or prime:boost vaccination regimens to elicit immunity towards multiple targets. In a study by Kim et al., the authors encoded H5 (A/Vietnam/1230/2004: H5N1), in addition to M2e as a potential pandemic vaccine candidate (224). Similar to using a wildtype PR8 infection, Ad5-H5/M2e protected mice from heterologous challenge with H5N2 virus, A/Aquaticbird/Korea/W81/2005. The vaccine elicited both HAI^+^ and NAb responses against the H5N2 virus, but impressively, also induced high titer stalk Abs (ELISA) which cross-reacted with the H1 stalk of cHA, cH9/1. In addition, H1-stalk reactive Abs elicited following immunization with Ad5-H5/M2e were sustained for 12 months. Vaccines that elicit broadly reactive Abs which are also durable would be a desirable outcome for a pandemic vaccine. In a separate manuscript, the authors also demonstrated that this vaccine was superior to Ad vaccines expressing either Ag alone when administered *i.n.* (225). Again, the Ad5-H5/M2e vaccine elicited robust stalk-specific Abs and was capable of protecting from heterosubtypic H1N1 challenge following a single immunization.

Alternatively, the use of bivalent Ad vaccines encoding more than one avian HA, or combinations of Ads expressing different HAs, has been investigated as a strategy to induce protective immunity against multiple avian influenza viruses in mice (226). Vemula and colleagues engineered Ad5 vaccines encoding two H5 immunogens, or H7 and H9, and compared these head-to-head with matched monovalent Ad vaccines which expressed each HA individually, as well as NP derived from an avian H5 strain. The authors subsequently tested these vaccines in a multivalent formulation, whereby combinations of bivalent vaccines were evaluated (ie. Ad-H5+H5 with Ad-H7+H9). A monovalent vaccine expressing NP was also combined with these bivalent Ads on the basis that NP on its own can only confer partial protection, but could increase heterosubtypic protection from challenge with unrelated viruses. All vaccines elicited Ag-specific cellular (IFN-γ ELISpot and NP/HA pentamer detection of Ag-specific CD8^+^ T cells) and humoral immune responses (HAI^+^ NAb and ELISA binding Abs), with increased breadth of protection from challenge with distinct viruses observed for the bivalent or multivalent vaccine formulations. The authors also noted the presence of stalk Abs following Ad immunization with multi-subtype HAs (as measured by ELISA against stalk peptides, not intact protein) (226). They concluded that these Abs did not play a role in protection due to their inability to reduce viral lung titers. However, it is now well-established that stalk-reactive Abs can be non-neutralizing (36) and would therefore not always reduce early viral infection and replication the lung, but may contribute to viral clearance at later time-points through engagement of Fc-mediated effector functions.

Many published studies have focused on evaluating HA, NP or M2e as vaccine immunogens. However, PB1 has also been selected as an Ag. Despite its recent implications in broadly cross-reactive CD8^+^ T cells in humans, its ability to confer protection in mouse models has been disappointing. Uddbäck and colleagues constructed an Ad5-based vaccine encoding PB1 and compared it to a similar vaccine encoding NP (212). The authors determined that PB1 was intrinsically less immunogenic than NP, and attempted to increase immune recognition of PB1 by combining it with an innovative genetic adjuvanting approach in which it was tethered to murine invariant chain (described in previous sections), resulting in increased frequencies of PB1-specific CD8^+^ T cells, as detected by ICS flow cytometry. However, despite these high frequencies, the modified Ad-PB1 vaccine was less protective than Ad-NP in a H1N1 (A/Puerto Rico/8/34) challenge, due to reduced killing capacity by PB1-specific CD8^+^ T cells. These data cast doubt on its potential as a broadly cross-protective immunogen, although future studies in humans may enable a more comprehensive understanding of the protective capacity of cross-reactive T cells which recognize PB1.

As outlined above, the concept of trying to increase the immune recognition and/or breadth of vaccine Ags encoded by Ad vectors by using molecular or genetic adjuvanting approaches (213, 227–229), has been applied to avian influenza viruses (230). Alternatively, vaccine administration using different routes of administration can elicit distinct immunogenicity profiles, with mucosal immunization being particularly attractive for strategies aimed at protecting against pathogens with a tropism for mucosal sites (98). To overcome poor immunogenicity due to limited Ag recognition following oral delivery of Ad vaccines, Scallan and colleagues at Vaxart, Inc encoded H5 (A/Indo/05/2005), in addition to a dsRNA hairpin, which acts as a TLR3 stimulant to induce Type I IFNs and thereby adjuvant the immune response to H5 (vaccine known as ND1.1). They demonstrated that this approach did indeed improve humoral immune responses to H5 following oral immunization in mice, but the adjuvant did not offer further improvements following intramuscular (*i.m*) vaccination, which was already immunogenic (and far superior to oral administration of Ad-HA without the TLR3 adjuvant component). Protection from homologous challenge was confirmed in mice and in ferrets, although only partial survival (6/8) was observed in ferrets immunized with Ad-HA-dsRNA administered orally as compared with *i.m* immunization, which had 100% survival. It is well established that the route of vaccine administration can have an impact on the magnitude and phenotype of immune response. This is due to differences in the types of cells present at the immunization site, and subsequently the *in vivo* tropism of the Ad vector, along with numerous species-specific factors (98) which could have affected efficacy in the aforementioned ferret study. The ND1.1 vaccine candidate subsequently advanced into *Phase I* clinical trials (NCT01335347), and was reported to elicit some modest T cell responses to H5 (ELISpot), but no HAI active Abs were detected (231).

### Rare Serotype Human Ad Vaccines

Although Ad5 is currently under evaluation in several clinical trials as a vaccine against SARS-CoV-2 (i.e., NCT04313127, NCT04436471, and NCT04437875), its widespread use in humans may be hampered by pre-existing immunity, which could negatively impact the magnitude of immunity directed towards the encoded transgene Ags. To overcome this issue, several alternative Ad vectors which are known to have low seroprevalence in humans have been evaluated pre-clinically. One such vector is a species E Ad, HAdV-E4 (Ad4) which has been used extensively by the US military in a replication-competent oral formulation to protect against Ad4 respiratory illness, suggesting that it may also be immunogenic when used as a vehicle to deliver avian influenza virus HAs. With this in mind, Alexander and colleagues tested a replication-competent Ad4 vector in mice (*human Ads do not replicate efficiently in mice due to host species restrictions*) encoding H5 from A/Vietnam/1194/2004 inserted into the E3 region of the Ad genome (126). When administered *i.n*, this vaccine elicited H5-specific cellular and humoral immune responses, even in the face of pre-existing immunity to Ad4. In support of this, altering the route of vaccine administration, or increasing the vector dose has previously been shown to help overcome pre-existing anti-vector immunity (232). Importantly, the vaccine was capable of conferring 100% protection from homologous challenge, with sterilizing protection in the lung at an Ad dose of 10^9^ viral particles (vp).

### Non-Human Ad Vaccines

#### AdC7

Similar to the rationale for investigating rare species human Ad vectors, many investigators have evaluated Ad vaccines derived from NHP as avian influenza vaccines, on the basis that these vectors have low seroprevalence in humans. Many promising vectors which have been developed were isolated from chimpanzees, which cluster phylogenetically with species E human Ads. A chimpanzee Ad vector AdC7 (*also known as ChAd7, SAd24, Pan7*) was engineered to encode NP from A/Puerto Rico/8/34 on the basis that the high conservation of NP could facilitate heterosubtypic protection from challenge with H5N1 avian influenza strains (127). When compared with Ad5-NP in mice, AdC7 elicited similar frequencies of IFN-γ^+^ CD8^+^ T cells, but the AdC7 vector appeared to elicit greater frequencies of polyfunctional CD8^+^ T cells (i.e., double or triple positive cytokine secretion). However, Ad5-NP still elicited superior, albeit partial, protection from challenge with two distinct H5N1 viruses, A/Vietnam1203/04 and A/Hong Kong/483/97. Another study by Cheng and colleagues evaluated the AdC7 vaccine encoding full length H5 (A/Chicken/Henan/12/2004) as the encoded transgene in a homologous prime:boost immunization regimen in mice with a dose of 5x10^10^vp (233). Ag-specific CD8^+^ T cell responses were detected following the prime (HA tetramer staining, and ICS), but these were not expanded upon boost, unlike HAI^+^ Abs which were not detected following prime, but reached titers of >1:125 following boost immunization. This AdC7-H5 vaccine conferred 100% protection from homologous lethal challenge, with no morbidity (weight loss) and minimal lung pathology. The authors also demonstrated that protection was Ab-mediated by performing passive transfer with immune sera prior to challenge.

#### AdC68

Xie and colleagues used consensus-based sequence selection and prediction of CD8^+^ T cell epitopes from six conserved IAV proteins, M1, M2, NP, PA, PB1, and PB2, to design a heterologous transgene Ag for incorporation into chimpanzee Ad vector, AdC68 (128) (*also known as ChAdV-68, ChAd68, SAd25, Pan9, ChAdOx2*) (98). When tested in a heterologous prime:boost regimen in mice with two DNA immunizations delivered *i.m*, AdC68 elicited robust cellular immune responses (IFN-γ ELISpot and ICS) and conferred complete protection from sub-lethal challenge with H7N9 A/Shanghai/4664T/2013. Although the authors also evaluated vaccine efficacy following *i.n* immunization with AdC68 and lethal H7N9 challenge, these results are complicated by the fact that they also used an additional vaccinia boost, making it difficult to evaluate the contribution of the Ad vector to protection. In a separate study, Zhou et al., inserted the conserved M2e epitope into hypervariable regions (HVR) within the AdC68 major capsid protein, the hexon (218). The hexon of Ads have a number of flexible loops (i.e., HVRs), which are exposed on the surface of the virion and have been shown previously to tolerate the insertion of targeting ligands or vaccine Ags (216, 234, 235). Following a head-to-head comparison of different constructs, including hexon-modified Ads encoding M2e and NP, the authors determined that modification of HVR1 was optimal for eliciting M2e-specific Abs. The HVR1-modified Ad vectors with or without the transgene were superior to other constructs in conferring protection following challenge with H1N1. However, vaccine efficacy was not evaluated for avian influenza challenge viruses. A subsequent study developed AdC68 encoding H7 HA (A/Zhejiang/DTID-ZJU01/2013) and tested its immunogenicity and efficacy in mice (236). The authors demonstrated that a single-shot at a dose of 5x10^10^vp was sufficient to elicit virus-specific NAb and T cell responses (ICS), and provide 100% protection from challenge with a heterologous H7N9 virus (A/Shanghai/4664T/2013).

#### ChAdOx1

A chimpanzee Ad (ChAd) platform, ChAdOx1, has also been evaluated pre-clinically, and has advanced to clinical trials as a vaccine for IAV and SARS-CoV-2 (67, 68, 113, 237). Using ChAdOx1 encoding NP+M1 or H7 HA in a prime:boost, or co-administration approach in mice, Tully and colleagues detected Ag-specific immune responses (IFN-γ and IgG ELISpot), as well as NAbs against H7 (129). However, all challenge experiments included an MVA boost immunization, so it is difficult to ascertain the protective efficacy of the ChAdOx1 vaccine platform from these studies. Subsequently, a collaborative effort between investigators in the US and UK tested ChAdOx1 vectors encoding cHA immunogens, in which the HA stalk was derived from H3 but the HA head domain was from an exotic HA strain, in addition to the NP+M1 fusion Ag (131). Again, the ChAdOx1 vaccine was not evaluated as a standalone vaccine, but in a prime:boost regimen with a modified vaccinia Ankara (MVA) boost. Various regimens were capable of conferring protection against challenge with three different G2 viruses: H3N2, H10N8 and H7N9. However, three sequential immunizations using viral vectors encoding cHAs (ChAd-MVA-cHA protein+adjuvant) were required to elicit strong G2 cross-reactive Ab responses. This would not be ideal in a rapidly evolving pandemic situation, where a single-shot vaccine which elicits robust and rapid cross-reactive immunity would be preferable. However, a subsequent prime:boost study in ferrets using the ChAdOx1 and MVA vaccines encoding cHAs (i.e., cH14/e or cH15/3) as well as vectors containing NP+M1, elicited Abs which cross-reacted with H3, H7 and H10 HAs, including H3 stalk-reactive Abs. This translated to reductions in viral titers in the respiratory tract of ferrets including nasal turbinates, the olfactory bulb and trachea (130). As ferrets are a very relevant animal model for the study of influenza vaccine efficacy, these data suggest that cHA-based immunization approaches, in combination with conserved Ags such as NP+M1, may be a promising approach to elicit broad immunity to IAVs in humans.

#### PAV-3

A promising platform based on a porcine Ad vector, PAV3-H5, with low seroprevalence in humans has also been developed as a vaccine for H5 avian influenza virus, encoding H5 from A/Hanoi/30408/2005 (132). When tested in mice, this vector elicited equivalent, or in some cases superior, humoral immune responses to H5 when compared with an Ad5-H5 control, and HAI^+^ Abs were sustained to higher levels one-year post-immunization. In addition, PAV3-H5 elicited more rapid cellular immunity (IFN-γ ELISpot and ICS) than Ad5-H5 prompting the investigators to assess virus challenge at early time-points post-immunization (D8 and D10), where they observed that survival following PAV3-H5 was superior to Ad5-H5. Vaccine efficacy with PAV3-H5 was also improved relative to Ad5-H5 when mice were challenged with homologous virus 28-day, or 1-year post-immunization.

#### BAdV-3

Similarly, another rare serotype vector based on a bovine Ad has been described by the Mittal laboratory (133). Again, the authors performed a head-to-head comparison with Ad5, known to be a potently immunogenic vector and as such represents a valuable benchmark for comparing the potency of novel Ad vaccine platforms (98). Ad5- and BAdV-3 vectors were engineered to encode H5 from A/Hong Kong/156/97, and vectors were evaluated in a dose de-escalation study in mice following *i.n* or *i.m* administration. Ab responses following *i.m* vaccination with BAdV-3 or Ad5 concluded that these platforms had similar immunogenicity. However, when vaccine was administered *i.n*, Ab responses to H5 were increased for the BAdV-3 vector, and this was particularly notable at low vector doses. Importantly, in addition to eliciting increased numbers of HA-specific CD8^+^ T cells relative to Ad5 (IFN-γ ELISpot), the novel BAdV-3 vector also elicited higher levels of IgA in the lung and nasal washes, as measured by ELISA. Impressively, this translated into sterilizing protection from viral infection of the lung following heterologous challenge with A/Vietnam/1203/2004, even at vaccine doses as low as 10^6^ plaque-forming-units (PFU). In contrast, the lowest dose of Ad5-H5 which conferred comparable protection in the lung was 3x10^7^ PFU. These data suggest that the BAdV-3 platform is a promising vaccine candidate for mucosal delivery, which would be well-suited to the development of vaccines for respiratory pathogens. In addition, the capacity to elicit robust immunogenicity, superior to that of Ad5, at low doses would make this platform very attractive for pandemic preparedness, potentially allowing for dose-sparing without loss of potency, as well as reducing the cost-per-dose for manufacturing.

### Clinical Trials

In addition to extensive evaluation in pre-clinical animal models, Ad-based vaccines for avian influenza have advanced into clinical trials in humans, using oral, *i.n* or *i.m* administration. The justification for administration *via* oral or *i.n* vaccination, is to stimulate mucosal immunity in the respiratory tract, the natural site of influenza virus infection. The earliest studies included a randomized *Phase I* trial (NCT01335347), in which 54 healthy subjects were administered orally with a non-replicating Ad5 vaccine encoding H5 (A/Indo/05/2005) and a dsRNA TLR3 ligand as a molecular adjuvant, formulated in a hypromellose capsule (231). Vaccinees were assigned into 10^8^, 10^9^, and 10^10^ infectious units (IU) groups, and compared with the placebo group. Four weeks following the prime immunization, 12 of 18 subjects in the intermediate group were given 10^9^ IU vaccine boost. Vaccines were well-tolerated with no adverse events reported over grade 1 severity. However, HAI^+^ Abs, an important current correlate of protection for influenza vaccines, were not detected. In addition, although the authors reported dose-dependent increases in cellular immunity (IFN-γ ELISpot) when compared to placebo group or pre-vaccination levels, these were very low level, suggesting that oral administration of non-replicating Ad5-based vectors, is not optimal in inducing immunity against influenza virus HA.

Subsequent studies tested an alternative Ad vector species E, HAdV-E4, using a similar oral capsule administration (100). However, this study selected a replication-competent Ad4 vaccine, rather than non-replicating. The rationale for choosing replication-competent Ad4 is based on its exemplary safety profile and historical use in the US military as a vaccine against Ad4 respiratory illness. To evaluate its potential as a vaccine delivery platform for avian influenza virus, the authors designed a randomized, multicenter, *Phase I* clinical trial to test PaxVax Ad4-H5-VTN (encoding H5 from A/Vietnam/1194/2004) in 166 subjects, aged 18–40. Vaccinees were assigned to one of five cohorts, each receiving three immunizations of a set dosage. Vaccine groups were administered doses ranging from 10^7^ to 10^11^vp, compared to a placebo group. Although all vaccines were well-tolerated, similar to findings with the oral Ad5 vaccine, Ad4-H5-VTN HA seroconversion as determined by HAI, MN, and GMT was low and was similar to placebo group. The authors propose that the poor inherent immunogenicity of avian influenza virus HAs, or cellular tropism of the Ad4 vector (currently unknown) following oral delivery may have contributed to the sub-optimal Ab responses, as it is well established that the latter can impact on the potency of Ad vaccines (98).

Upon completion of the 3-vaccine regimen, 105 subjects elected to take part in a follow-up study. Sub-study subjects were then boosted with 90μg of inactivated parenteral H5N1 vaccine. Interestingly, following boosting with the inactivated H5N1 subvirion vaccine, seroconversion (as determined by a four‐fold rise in baseline HAI^+^ titer) and seroprotection (as measured by HAI titres ≥40) in vaccine groups were noticeably increased when compared to placebo group. This was best seen in the 10^11^vp cohort which exhibited 100% seroconversion and 89% seroprotection by HAI, compared with placebo group displaying 33 and 14%, respectively. H5-specific cellular responses in this study were similar to those seen in *i.n* LAIV. From these findings, it is possible that using Ad vaccines as a prime may enable improvements in Ab and cellular immunity for Ags which are intrinsically poor in terms of immunogenicity.

Considering the poor humoral immunity following oral vaccination with either Ad5- or Ad4-based vaccines, and implications that the route of administration may have negatively impacted on the induction of robust humoral immunogenicity, *Phase I* clinical trials (NCT00755703 and NCT01806909) were initiated to evaluate *i.n* administration of the aforementioned Ad5 and Ad4 vectors for influenza. These studies collectively aimed to monitor safety and immunogenicity in healthy adults from ages 18–49, but formal published findings have not yet been reported. A more recent clinical study in humans (NCT01443936) tested the replication-competent PaxVax vaccine Ad4-H5-VTN (A/Vietnam/1194/2004) by tonsillar, *i.n*, or oral route (238). This vaccine was administered to 56 healthy individuals, aged 18–49, with doses ranging from 10^3^ and 10^8^vp for the tonsillar or *i.n* route, and a dose of 10^10^vp administered orally. Ad4 seroconversion was seen in tonsillar and *i.n* groups at doses of >10^4^vp. Although overall serum neutralization by MN assay against H5 was modest, Abs with broadly neutralizing activity and potency were detected in peripheral memory B cell populations. Importantly, although not reflected at the serum level, immunization with the replication-competent Ad4-H5 vector induced prolonged increases in somatic hypermutation (SHM), and subsequently increased Ab potency against H5 for several months. In addition, numerous novel mAbs were identified, including one stalk-reactive Ab belonging to a new multidonor class of Abs. Ad vaccines are well-established in animal models to facilitate sustained transgene Ag expression (98, 122, 239, 240), which, although not formally investigated in this study, may have contributed to the prolonged evolution of Ag-specific B cell responses. However, these data highlighted the importance of considering sustained B cell evolution as a valuable parameter when evaluating the success of various vaccine platforms. Better understanding the kinetics and significance of this process may enable the design of optimal vaccine platforms or immunization regimens designed to elicit broad and durable protective immunity. Although promising, some disadvantages in using replication-competent Ads is the induction of anti-vector immunity and its possible competition with transgene Ag. In addition, the use of replication-competent vaccines, including Ad4, are contraindicated for use in young children, pregnant women, the elderly and immunocompromised individuals, thereby limiting its widespread use as a pandemic vaccine to protect vulnerable, at-risk groups.

In addition to human Ads, vectors derived from NHPs have also been evaluated in humans. One candidate vaccine is a species E chimpanzee Ad vector encoding conserved IAV Ags NP and M1, with the aim of stimulating cross-reactive and heterosubtypic cellular immunity. A first-in-human *Phase I* dose-escalation trial to evaluate the immunogenicity of non-replicating ChAdOx1-NP+M1 (Ag sequence from H3 A/Panama/2007/99) following *i.m* immunization was carried out with 15 subjects in a 3 + 3 study model (67). Overall, the vaccine was well tolerated in volunteers, allowing the progression to the highest dose of 5x10^10^vp. However, two of the three vaccinees in this group experienced local and systemic reactions, concluding that this dose was not ideal for a prophylactic vaccine. All groups displayed increases in T cell responses, most peaking day 14 post-vaccination. In addition, a sub-set of participants in the 5x10^10^vp group received a heterologous boost with 1.5x10^8^ PFU of MVA-NP+M1, resulting in all three subjects displaying an increase in Ag-specific T cells (IFN-γ ELISpot); responses which were elevated for 8 weeks post-boost. This first-in-human clinical trial was subsequently extended in a *Phase I*, randomized, multicenter study trial to evaluate a heterologous two-dose vaccination regimen using ChAdOx1-NP+M1 and MVA-NP+M1 *i.m* in 49 healthy subjects, aged 18–46, and 24 subjects, age 50 and over. The vaccine regimen proved safe and immunogenic for both younger and older adults. In young adults, the MVA/ChAdOx1 regimen, regardless of the time interval between first and second immunization, elicited T cell responses which remained elevated compared to the ChAdOx1/MVA regimen. In addition, a single vaccination with MVA displayed significantly higher fold-increase and peak immune responses compared to a single vaccination with ChAdOx1 vaccine in young adults. However, all groups following first vaccination in both younger and older adults, compared to baseline, displayed significantly elevated T cell responses to NP and M1 for up to 18 months (ELISpot and ICS). The two-dose heterologous vaccination regimens significantly increased the frequency of cross-reactive T cell responses to NP and M1 (ICS). This study confirmed the ability for viral vector vaccines to elicit sustained T cell responses to conserved Ags which have the potential for heterosubtypic cross-reactivity.

## Challenges Facing the Advancement of Adenoviral Vaccines

### Pre-Existing Immunity

The development of vaccines based on Ad5 has waned in the last two decades, largely due to the seroprevalence of anti-Ad5 Abs in humans (which can differ geographically) and the potential for anti-vector Abs to limit vaccine efficacy (241). This topic has once again become the subject of debate, as a result of several Ad vectors undergoing evaluation as vaccines for SARS-CoV-2. Indeed, in clinical trials to evaluate an Ad5-based vaccine in humans, high-level (>1:200) pre-existing Abs to Ad5 (as well as increased age), compromised seroconversion to the encoded Ad5-delivered SARS-CoV-2 spike (109, 242). Interestingly, reactogenicity was reduced in older adults, and those with high pre-existing anti-vector Abs, suggesting that booster immunizations to overcome limited immunogenicity might be tolerated in these groups. However, in our opinion, the use of an alternative Ad serotype such as the Ad26 or ChAdOx1 vector, or a completely different vaccine platform as a booster would be preferable in eliciting an optimal immune response. Alternatively, increasing the interval between re-administration of the same Ad vector, or altering the route of administration may help to overcome the effects of anti-vector immunity, as demonstrated in mice (232, 243, 244).

Additionally, discouraging data from the Merck STEP vaccine trial: a HIV vaccine trial using Ad5 encoding *gag*, *pol* and *nef*, administered to men and women across the Americas, Caribbean and Australia (245), also dampened enthusiasm for Ad5-based vaccines. The STEP trial was halted after the first interim analysis, as the pre-determined, non-efficacy boundaries were reached. Post-hoc analyses revealed a trend towards increased HIV acquisition in vaccinated males (24/522 males), compared to the placebo group (20/536 males) (246). This was associated with an increased hazard ratio in men who were uncircumcised and had high pre-existing Ad5 Abs, in addition to being linked to specific sexual practices (245).

More recently, case-control study of two cohorts (*n* = 889 total) at elevated risk of HIV-1 infection showed no association between Ad5 seropositivity and incidence of HIV-1 infection (247). Furthermore, a larger case-control study (*n* = 1570) showed no association between pre-existing Abs to seven Ad serotypes (Ad1, -2, -6, -26, -35, and -48) and acquisition of HIV-1 infection across three HIV-1 vaccine efficacy trials: the VAX003 and VAX004 trials of non-adenoviral vectored vaccines, and the Merck Ad5 STEP study (248). With pre-existing Ad5 antibodies alone seemingly not a risk factor, it could be argued that the trend towards increased HIV acquisition in the STEP trial was a product of testing a non-efficacious vaccine (which lacked an Env antigen), in extremely specific cohorts with high HIV transmission. Indeed, as outlined above, pre-clinical and clinical vaccine development using Ad vectors has successfully continued. With this in mind, the possibility for repeated administration of Ad-based vaccines is supported by the large range of novel vectors to choose from, many of which have been, or are currently being vectorized (98). In addition, it is possible to make genetically chimeric Ad vectors, in which the major targets for type-specific anti-vector Abs (the fiber, or hexon) (249) are swapped for corresponding regions from rare Ad viruses (249).

### Manufacturing Capacity

One further challenge, not limited to Ad vectors, is matching clinical grade vaccine production output with demand. Further to this, there can be differences in the manufacturing characteristics of distinct Ad serotypes (i.e., growth to high titers, genetic stability). The SARS-CoV-2 pandemic and urgent need for rapid production of a safe and effective vaccine has highlighted the importance of investing in scalable vaccines which are well-suited to stockpiling. As previously stated, Ad vectors in general have a number of beneficial attributes in terms of their potential for thermostabilization and cold-chain free storage requirements (98). However, outside of SARS-CoV-2 investment, traditional manufacturing processes for Ad vectors have not previously been considered cost-effective or commercially viable for global scale production (250). Vellinga and colleagues have summarized these issues and have highlighted strategies to improve this in an excellent review from 2014 (250). In terms of pandemic preparedness and the development of vaccines with immunological breadth to protect against antigenically drifted, or shifted viruses such as avian influenza virus, Ad vectors are ideal and are therefore a worthy investment as a tool to combat emerging viral infections (241).

### Summary

Advances in innovative immunogen design will undoubtedly enable the development of optimized vaccine platforms capable of eliciting breadth of reactivity, in addition to durability. For example, the use of computationally designed immunogens such as COBRA (251) or immunogens designed using Epigraph algorithms (252) may help to increase breadth across multiple Ags, against T cell epitopes and/or discontinuous B cell epitopes. Pre-clinical validation of these immunogens could also help to identify as-yet-undefined epitopes which play important roles in heterosubtypic protection against novel influenza subtypes

Significant advances have been made in developing novel HA immunogens with the aim of boosting immune responses against the highly conserved but immunosubdominant HA stalk. The HA head is immunodominant and antigenically variable, and in general, Abs elicited against this domain are strain-specific. This is a major factor contributing to the requirement to reformulate conventional IIV-based vaccines on an annual basis. However, highly conserved, broadly neutralizing epitopes do also exist in the HA head domain (253–255). Although Abs to these epitopes are rare and appear to be poorly elicited by seasonal vaccines (256), head-specific mAbs capable of neutralizing multiple IAV subtypes have been isolated from animals (257, 258) and humans (256, 259). Many of the target epitopes are located proximal to the RBS, and in some cases the mAbs use molecular mimicry of the HA surface receptor SA (256). However, anti-HA head mAbs with broad reactivity have also been identified which recognize epitopes distinct from the RBS (260–262). Therefore, conserved HA head epitopes could represent a novel target for next-generation influenza virus vaccine design. The use of structural biology techniques and alternative vaccine platforms may provide additional insight, and enable the improved induction of responses directed towards these unusual, or occluded epitopes, in a manner superior to conventional vaccine platforms.

In recent years, the influenza vaccine field has invested a significant amount of time in trying to better understand the differences between immunity elicited through immunization with different platforms, and natural infection. Evidence is growing that the primary influenza virus exposure in early life, or “*immunological imprinting*”, can have a major impact on subsequent responses and susceptibility to infection with G1 or G2 IAVs in later life (263, 264). This is an important consideration when developing broad, or universal influenza virus vaccines, as it is unclear if such a vaccine should elicit equivalent immunity to G1 and G2 HAs simultaneously, and if immunization should ideally be prior to primary natural infection, in very early childhood. The foundations to support these major questions are currently being addressed by large cohort studies in humans, comparing populations with low vaccine coverage versus those with annual seasonal influenza vaccine programs (167, 265). Substantial funding investment to support the development of a universal influenza virus vaccine in recent years will undoubtedly have a positive impact on vaccines which elicit protective immunity, extending to the design of vaccines to protect against emerging avian influenza viruses.

## Author Contributions

SB and LK—wrote initial draft. LC and CB—wrote manuscript and oversaw final edits. LC—supervision. LC—funding. All authors contributed to the article and approved the submitted version.

## Funding

LC is funded in part by National Institute of Allergy and Infectious Diseases (NIAID) R21AI146529, by Centers of Excellence for Influenza Research and Surveillance (CEIRS) contract HHSN272201400008C and by a small grant awarded by the Royal Society for Tropical Medicine and Hygiene (GR000550). CB is funded by a Wellcome Trust ISSF fellowship. 

## Conflict of Interest

The authors declare that the research was conducted in the absence of any commercial or financial relationships that could be construed as a potential conflict of interest.
